# Redefining Obesity Risk for Heart Failure: Incorporating Morbidity Into the 2024 EASO Framework Definition of Obesity

**DOI:** 10.1002/dmrr.70227

**Published:** 2026-09-07

**Authors:** Liang Tang, Xianming Tang, Zhenhua Xing, Chenkai Wu

**Affiliations:** ^1^ Department of Cardiovascular Medicine Second Xiangya Hospital Central South University Changsha Hunan China; ^2^ Department of Emergency Medicine Second Xiangya Hospital Central South University Changsha Hunan China

**Keywords:** body mass index, European Association for the Study of Obesity, heart failure, obesity

## Abstract

**Background:**

Conventional BMI‐based obesity classification fails to capture the metabolic heterogeneity underlying obesity‐related heart failure (HF) risk. We evaluated the 2024 European Association for the Study of Obesity (EASO) framework—which integrates central adiposity with medical, functional, and psychological morbidity—to refine HF risk stratification.

**Methods:**

Among 449,550 UK Biobank participants (median follow‐up 15.5 years), we applied three obesity definitions: (1) BMI ≥ 30 kg/m^2^; (2) the EASO framework, which retains the normal‐weight category (BMI 18.5–< 25 kg/m^2^) and reclassifies individuals with BMI 25–< 30 kg/m^2^ into ‘EASO overweight’ (WHtR < 0.5 and/or absence of morbidity) or ‘EASO new obesity’ (WHtR ≥ 0.5 plus ≥ 1 morbidity), while those with BMI ≥ 30 kg/m^2^ are classified as BMI obesity regardless of morbidity status; and (3) an extended EASO framework that additionally stratifies individuals with BMI ≥ 30 kg/m^2^ by morbidity status. Morbidity was defined per the 2024 EASO consensus criteria as the presence of ≥ 1 medical, functional, or psychological conditions associated with excess adiposity. Cox proportional hazard models estimated hazard ratios (HRs) for incident HF.

**Results:**

The EASO framework reclassified 15.6% of conventionally overweight individuals as obese, increasing the obesity prevalence from 24.5% to 40.1%. Compared with normal‐weight individuals, the EASO overweight group (BMI 25–< 30 kg/m^2^ without central adiposity or morbidity) exhibited the lowest HF risk (HR 0.84, 95% CI 0.80–0.89), confirming that overweight per se confers no excess risk in the absence of morbidity. By contrast, EASO new obesity (BMI 25–< 30 kg/m^2^ with central adiposity and ≥ 1 morbidity; HR 1.32, 95% CI 1.25–1.39) demonstrated that morbidity presence—not BMI threshold—drives elevated HF risk within the overweight range. BMI obesity (BMI ≥ 30 kg/m^2^) yielded the highest summary HR (1.62, 95% CI 1.53–1.71), reflecting its compositional enrichment with high‐morbidity individuals rather than superior risk discrimination. In the extended framework, morbidity status produced a consistent vertical upward shift in HF risk across all adiposity categories, establishing phenotypic characterisation beyond anthropometric thresholds as the key determinant of HF vulnerability.

**Conclusions:**

The EASO framework refines HF risk stratification by uncovering morbidity‐driven vulnerability within the overweight range that BMI alone obscures. The extended framework further demonstrates that morbidity status—rather than anthropometric thresholds—is a primary modifier of HF risk across all adiposity categories, supporting a shift from purely anthropometric toward phenotype‐based obesity classification in cardiovascular risk assessment.

## 1 Introduction

Obesity has emerged as a major global health challenge and is a well‐established independent risk factor for heart failure (HF) [1, 2, 3]. However, despite its substantial clinical impact, obesity—when defined solely by body mass index (BMI)—is not universally recognized as a disease entity, and no single consensus definition of obesity as a ‘disease’ currently exists [4]. Importantly, reliance on BMI alone has important limitations. As a surrogate of overall adiposity, BMI fails to capture heterogeneity in fat distribution, obesity‐associated comorbidities, and underlying metabolic health, leading to potential misclassification of individuals at risk for obesity‐related conditions [5, 6]. In response to growing recognition that excess body weight alone inadequately reflects obesity‐related health risk, the European Association for the Study of Obesity (EASO) recently proposed a revised framework for obesity diagnosis and management that integrates BMI, central adiposity, and obesity‐related morbidity [7]. Within this framework, obesity is conceptualised not solely as excess body weight but as increased adiposity accompanied by clinically relevant health consequences. Operationally, obesity is defined as a BMI ≥ 30 kg/m^2^, or a BMI between 25 and < 30 kg/m^2^ in the presence of central adiposity (waist‐to‐height ratio [WHtR] ≥ 0.5) and at least one medical, functional, or psychological complication [7].

In a recent U.S. analysis using NHANES data, individuals classified as EASO new obesity exhibited a higher risk of all‐cause mortality than those categorised as EASO overweight, providing initial validation of the framework [8]. Notably, although mortality risk increased across EASO categories, individuals with EASO new obesity demonstrated a risk profile comparable to normal weight and substantially lower than that observed in BMI obesity (BMI ≥ 30 kg/m^2^) [8]. Importantly, morbidity status appeared to be a key determinant of risk within both EASO overweight and EASO new obesity groups, suggesting that obesity‐related complications, rather than BMI alone, may reflect underlying pathophysiological processes driving adverse outcomes. Despite these insights, several knowledge gaps remain. The EASO framework primarily refines risk stratification within the overweight range, while potential heterogeneity among normal‐weight and obese individuals—particularly with respect to morbidity status—has received limited attention [7]. Moreover, prior analyses have focused exclusively on all‐cause mortality, leaving the relationship between EASO‐defined obesity and HF, one of the most clinically consequential obesity‐related outcomes, largely unexplored [8].

To address these gaps, we leveraged data from the UK Biobank, a large prospective population‐based cohort, to comprehensively evaluate the EASO obesity framework. Our objectives were threefold: first, to quantify the impact of the EASO definition on obesity prevalence in a large‐scale population; second, to examine its associations with HF risk; and third, to explore how morbidity modifies these associations across the full BMI spectrum.

## 2 Methods

### 2.1 Study Population

The UK Biobank is a nationwide, population‐based prospective cohort established to investigate determinants of health and disease in midlife and older adults. From 2006 to 2010, over 500,000 individuals aged 40–69 years were recruited from socioeconomically and ethnically diverse urban and rural regions across England, Scotland, and Wales. Recruitment was conducted through 22 assessment centres, with invitations mailed to National Health Service–registered residents living within approximately 25 miles of a participating centre [9]. At enrolment, participants underwent standardized physical examinations, provided biological samples, and completed extensive questionnaires capturing demographic information, lifestyle factors, and medical history. The study received ethical approval from the North West Multi‐Centre Research Ethics Committee (11/NW/0382) and adhered to the principles of the Declaration of Helsinki. Participant data were anonymised prior to analysis, and individuals did not contribute to the study design, data collection, or analytical processes. Comprehensive study documentation, including protocols and data dictionaries, is available at http://www.ukbiobank.ac.uk/.

### 2.2 Obesity Definitions

Obesity was defined using three complementary approaches [1]: BMI‐based classification [2], the 2024 EASO framework, and [3] an EASO‐based classification further stratified by morbidity status. Participants were excluded if they had missing BMI or WHtR data, BMI < 18.5 kg/m^2^, or a history of cancer at baseline. Under the BMI‐based approach, participants were categorised as normal weight (BMI 18.5–< 25 kg/m^2^), overweight (BMI 25–< 30 kg/m^2^), or obese (BMI ≥ 30 kg/m^2^). According to the EASO framework, participants were classified into four mutually exclusive categories: normal weight (BMI 18.5–< 25 kg/m^2^); EASO overweight (BMI 25–< 30 kg/m^2^ with WHtR < 0.5 and/or absence of medical, functional, or psychological morbidity); EASO new obesity (BMI 25–< 30 kg/m^2^ with WHtR ≥ 0.5 and ≥ 1 morbidity); or BMI obesity (BMI ≥ 30 kg/m^2^, regardless of morbidity status) [7]. For the morbidity‐stratified EASO‐based classification, both normal weight and BMI obesity were further subdivided by morbidity status, yielding six mutually exclusive groups: normal weight without morbidity, normal weight with morbidity, EASO overweight, EASO new obesity, BMI obesity without morbidity, and BMI obesity with morbidity.

### 2.3 Study Outcomes and Morbidity Definitions

Outcomes and morbidity status were identified using the UK Biobank ‘First occurrences’ data fields (Field IDs), with event dates used to distinguish incident events from conditions present prior to enrolment. The primary study outcomes were HF (Field ID: 131355). Follow‐up period extended from baseline to the first occurrence of an outcome event (Field ID: 131354) or until 30 September 2024, whichever occurred first. Morbidity status used for stratification was restricted to conditions documented before baseline and included hypertension, cardiovascular disease (CVD; including angina, myocardial infarction, or stroke), diabetes, arthritis, chronic kidney disease (CKD), chronic obstructive pulmonary disease (COPD), and depression. CKD was defined as an eGFR < 60 mL/min/1.73 m^2^ at baseline, calculated using the CKD‐EPI equation. Arthritis was identified using UK Biobank Field ID 120000, while all other morbidities were ascertained through the ‘First occurrences’ fields, with diagnosis dates confirming presence prior to enrolment. Detailed mappings of outcomes and morbidities to Field IDs, coding algorithms, and derivation rules are provided in Supporting Information S1: Table S1.

### 2.4 Covariates

Baseline covariates were derived from questionnaires, physical examinations, laboratory assays, and linked health records collected at UK Biobank assessment centres. Demographic variables included age, sex (men or women), race (White or non‐White), and educational attainment (college or university vs. below college). Socioeconomic status was assessed using the Townsend Deprivation Index (TDI), an area‐level measure derived from national census data [10]. Lifestyle factors comprised smoking status (never, previous, current, or no answer) and alcohol consumption (never, previous, current, or no answer). Dietary habits were summarised using a diet score ranging from 0 to 4, reflecting adherence to key dietary recommendations: consumption of at least four tablespoons of vegetables per day, three pieces of fruit per day, fish intake at least twice weekly, and limiting processed meat intake to no more than twice per week [5]. Employment status was categorised as in employment, retired, or unemployed/no answer. Physical activity was quantified as total metabolic equivalent minutes per week (MET) based on baseline questionnaires. Clinical and anthropometric measures included systolic blood pressure (SBP), diastolic blood pressure (DBP), weight‐adjusted grip strength, waist circumference (WC), hip circumference, BMI, and WHtR. Laboratory biomarkers included fasting plasma glucose (FPG), serum lipids—high‐density lipoprotein (HDL), low‐density lipoprotein (LDL), and triglycerides (TG)—urate, creatinine, and C‐reactive protein (CRP).

### 2.5 Statistical Methods

Categorical variables were compared using chi‐square tests, whereas continuous variables were analysed using ANOVA or Mann–Whitney U tests, as appropriate. Participants with missing values in BMI, WHtR, or morbidity status were excluded from the analysis; missing values in all other covariates were handled using multiple imputation. Associations between obesity categories and HF risk were evaluated using Cox proportional hazards models. In the primary analysis, obesity categories were defined according to the 2024 EASO framework, with normal weight (BMI < 25 kg/m^2^) as the reference. HRs and 95% CIs were estimated for EASO overweight, EASO new obesity, and BMI obesity (BMI ≥ 30 kg/m^2^). In the secondary analysis, EASO categories were further stratified by morbidity status, using normal‐weight participants without morbidity as the reference. This strategy enabled direct comparisons across normal‐weight participants with morbidity, EASO overweight, and EASO new obesity, while BMI obesity was also examined separately in participants with and without morbidity. Model 1 was unadjusted. Model 2 adjusted for age, sex, race, TDI, MET, diet score, smoking, alcohol intake, education, employment, grip strength, and AF. Model 3 was further adjusted for HDL, LDL, FPG, SBP, DBP, and eGFR. To minimise bias in HF risk estimation, competing risk models were employed, with all‐cause mortality treated as a competing event. Restricted cubic splines were used to model the continuous association between BMI and incident HF risk, stratified by morbidity status, within a single Cox proportional hazards model incorporating a morbidity‐by‐spline interaction term (adjusted per Model 3). Hazard ratios were calculated relative to BMI = 25 kg/m^2^ among participants without morbidity, corresponding to the conventional normal weight–overweight boundary. To account for potential age‐related heterogeneity, analyses were stratified by age (≥ 56 years, median). To assess whether the elevated HF risk associated with EASO new obesity reflected the obesity reclassification itself rather than the independent effects of the constituent morbidities, we conducted a pre‐specified sensitivity analysis excluding hypertension and CVD from the morbidity definition, given that these are the two strongest established HF risk factors among the EASO‐specified conditions. All other analytical procedures were identical to the primary analysis. Additional subgroup analyses were conducted according to sex and race. All statistical analyses were conducted using R (version 4.4.0) and Stata (version 17; StataCorp, College Station, TX, USA).

## 3 Results

### 3.1 Study Population

**FIGURE 1:**
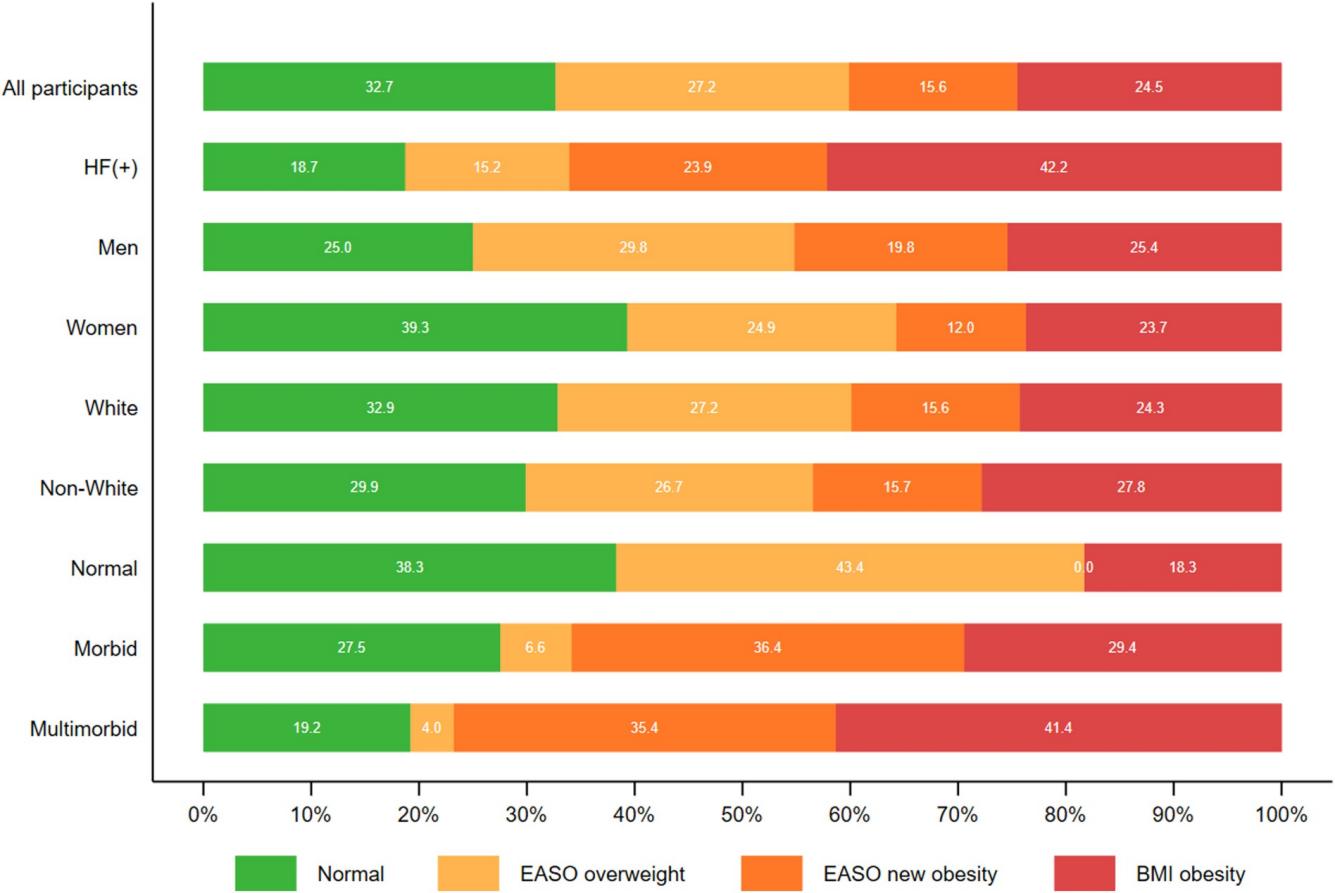
Distribution of the study population according to the 2024 EASO framework definition of obesity. EASO, European Association for the Study of Obesity.

Of the 501,991 participants in the UK Biobank, 489,328 had available BMI and WHtR data after excluding those with missing anthropometric measures or a BMI < 18.5 kg/m^2^. After additionally excluding 37,713 individuals with a history of cancer and 2111 with a history of HF, a total of 449,550 participants were included in the final analysis (Supporting Information S1: Figure S1). Based on BMI, 32.7% of participants were classified as normal weight, 42.8% as overweight, and 24.5% as obese. Applying the EASO criteria led to the reclassification of 15.6% of individuals previously considered overweight as obese, increasing the overall obesity prevalence to 40.1% (Figure 1). Among participants who later developed HF, 23.9% of those initially categorised as overweight were reclassified as obese, resulting in an obesity prevalence of 66.1%. Reclassification was more frequent in men than in women (19.75% vs. 12.03%) and did not differ substantially across ethnic groups (Figure 1). Participants who developed HF were older, predominantly male, and more often White, with higher TDI scores, greater prevalence of AF, and more current smokers. They had lower diet scores and alcohol consumption, were more likely to be retired, and had lower education levels. Total and central adiposity were higher, whereas physical activity and grip strength were lower. SBP, DBP, CRP, TG, and FPG were elevated, whereas HDL, LDL, and eGFR were reduced. Chronic conditions including hypertension, depression, COPD, diabetes, CVD, and CKD were more prevalent, whereas arthritis prevalence was lower (Table 1).

**TABLE 1:** Baseline characteristics of participants according to incident HF status.

Variable	Total population	Without HF	With HF	*p*‐value
*N*	449,550	433,497	16,053	
Age (years)	56.22 ± 8.11	56.01 ± 8.09	61.98 ± 6.23	< 0.001
Women (%)	241,368 (53.69)	235,541 (54.34)	5827 (36.30)	< 0.001
Race, white (%)	422,140 (93.90)	406,997 (93.89)	15,143 (94.33)	0.021
TDI	−1.31 ± 3.08	−1.33 ± 3.07	−0.72 ± 3.34	< 0.001
AF (%)	6128 (1.36)	4899 (1.13)	1229 (7.66)	
Smoking history (%)				< 0.001
Current	47,237 (10.51)	44,723 (10.32)	2514 (15.66)	
Previous	153,088 (34.05)	146,143 (33.71)	6945 (43.26)	
Never	246,974 (54.94)	240,504 (55.48)	6470 (40.30)	
No answer	2251 (0.50)	2127 (0.49)	124 (0.77)	
Diet scores	1.88 ± 1.05	1.88 ± 1.05	1.84 ± 1.06	< 0.001
Alcohol consumption (%)				< 0.001
Current	413,321 (91.94)	399,190 (92.09)	14,131 (88.03)	
Previous	15,479 (3.44)	14,516 (3.35)	963 (6.00)	
Never	19,617 (4.36)	18,718 (4.32)	899 (5.60)	
No answer	1133 (0.25)	1073 (0.25)	60 (0.37)	
Employment status (%)				< 0.001
In employment	245,670 (54.65)	241,001 (55.59)	4669 (29.08)	
Retired	130,916 (29.12)	122,688 (28.30)	8228 (51.26)	
Unemployed or no answer	72,964 (16.23)	69,808 (16.10)	3156 (19.66)	
Education levels (%)				< 0.001
College or university	51,034 (11.35)	49,770 (11.48)	1264 (7.87)	
Below	398,516 (88.65)	383,727 (88.52)	14,789 (92.13)	
BMI (kg/m^2^)	27.46 ± 4.73	27.37 ± 4.66	29.86 ± 5.73	< 0.001
WC (cm)	90.36 ± 13.33	90.04 ± 13.17	99.02 ± 14.60	< 0.001
WHtR	0.54 ± 0.07	0.53 ± 0.07	0.58 ± 0.08	< 0.001
MET (min/week)	2653.96 ± 2332.25	2656.23 ± 2332.12	2592.47 ± 2335.07	< 0.001
Grip strength (kg)	0.80 ± 0.25	0.80 ± 0.25	0.72 ± 0.26	< 0.001
DBP (mmHg)	82.28 ± 10.31	82.26 ± 10.28	82.95 ± 11.16	< 0.001
SBP (mmHg)	139.66 ± 18.93	139.41 ± 18.83	146.40 ± 20.21	< 0.001
Hip (cm)	103.43 ± 9.10	103.30 ± 9.00	106.82 ± 11.03	< 0.001
CRP (mg/L)	2.55 ± 4.10	2.50 ± 4.02	3.81 ± 5.69	< 0.001
HDL (mmol/L)	1.45 ± 0.35	1.45 ± 0.35	1.34 ± 0.35	< 0.001
LDL (mmol/L)	3.56 ± 0.84	3.57 ± 0.83	3.32 ± 0.90	< 0.001
TG (mmol/L)	1.74 ± 0.99	1.74 ± 0.99	1.92 ± 1.06	< 0.001
Urate (μmol/L)	309.17 ± 77.30	307.88 ± 76.69	343.94 ± 85.29	< 0.001
FPG (mmol/L)	5.11 ± 1.13	5.10 ± 1.09	5.50 ± 1.90	< 0.001
BMIP	−0.19 ± 0.98	−0.20 ± 0.98	−0.04 ± 0.98	< 0.001
eGFR (mL/min/1.73 m^2^)	105.24 ± 27.14	105.25 ± 26.96	104.77 ± 31.71	0.027
Chronic disease (%)				
Arthritis	50,435 (11.22)	49,159 (11.34)	1276 (7.95)	< 0.001
Hypertension	117,744 (26.19)	109,247 (25.20)	8497 (52.93)	< 0.001
Depression	36,927 (8.21)	35,349 (8.15)	1578 (9.83)	< 0.001
COPD	7081 (1.58)	6110 (1.41)	971 (6.05)	< 0.001
Diabetes	11,204 (2.49)	9597 (2.21)	1607 (10.01)	< 0.001
CVD	25,186 (5.60)	21,397 (4.94)	3789 (23.60)	< 0.001
CKD	7403 (1.65)	6789 (1.57)	614 (3.82)	< 0.001
Morbidity status (%)				< 0.001
Normal	256,336 (57.02)	251,348 (57.98)	4988 (31.07)	< 0.001
Morbid	193,214 (42.98)	182,149 (42.02)	11,065 (68.93)	< 0.001
Multimorbid	51,501 (11.46)	46,247 (10.67)	5254 (32.73)	< 0.001

Abbreviations: BMI, body mass index; BMIP, BMI polygenic risk score; COPD, chronic obstructive pulmonary disease; CKD, chronic kidney disease; CRP, C‐reactive protein; CVD, cardiovascular disease; DBP, diastolic blood pressure; EASO, European Association for the Study of Obesity; eGFR, estimated glomerular filtration rate; FPG, fasting plasma glucose; HDL, high‐density lipoprotein; LDL, low‐density lipoprotein; MET, metabolic equivalent of task; SBP, systolic blood pressure; TG, triglycerides; TDI, townsend deprivation index; WC, waist circumference; WHtR, waist‐to‐height ratio.

### 3.2 EASO Criteria and HF Risk

**FIGURE 2:**
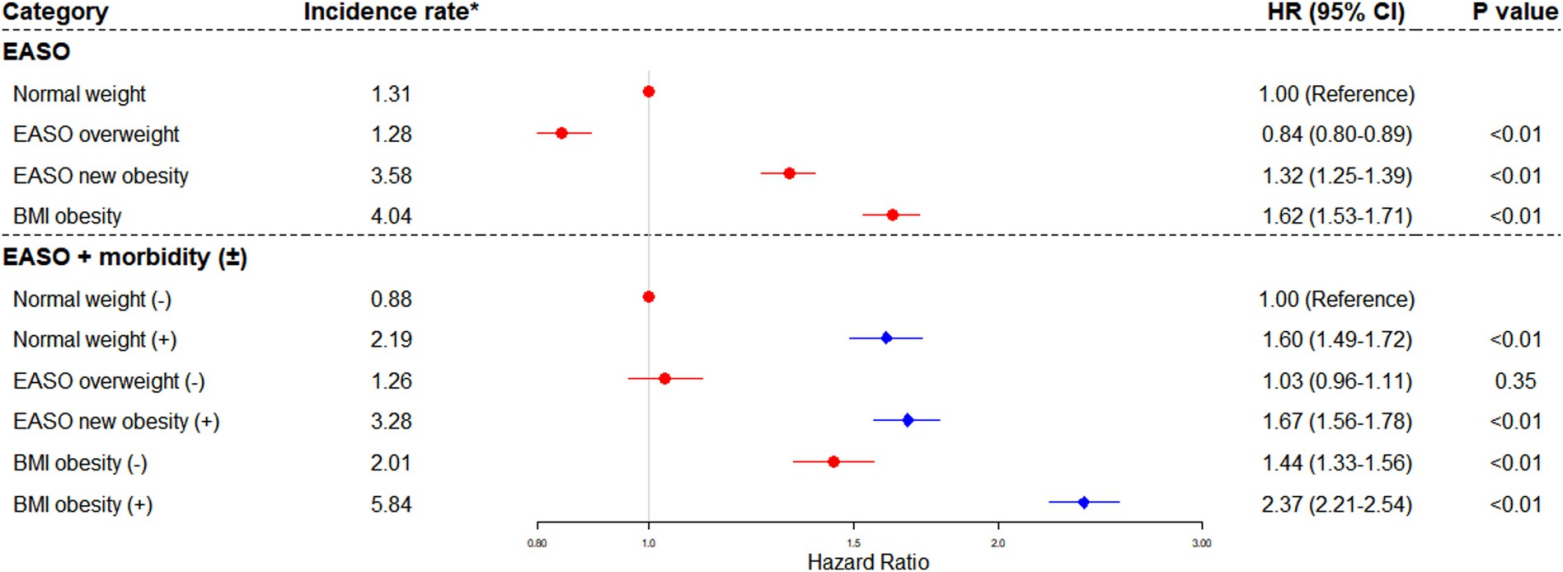
Associations between obesity categories and HF. Circles indicate groups without morbidity, and diamonds indicate groups with morbidity. Models were adjusted for Model 3. BMI, body mass index; EASO, European Association for the Study of Obesity; HF, heart failure; HR, hazard ratio. *Incidence rate per 1000 person‐years.

Over a mean follow‐up of 15.5 ± 1.7 years, 16,053 participants developed HF. In primary analysis using the EASO framework, compared with the normal‐weight reference group (BMI < 25 kg/m^2^), the EASO overweight group exhibited the lowest risk (HR 0.84, 95% CI 0.80–0.89), followed by EASO new obesity (EASO with morbidity) (HR 1.32, 95% CI 1.25–1.39), whereas BMI obesity had the highest risk (HR 1.62, 95% CI 1.53–1.71) (Table 2 and Figure 2). However, the normal‐weight reference group in this analysis is heterogeneous, encompassing individuals both with and without morbidity; this heterogeneity inflates its summary risk and may render the EASO overweight group artificially protective. Results were robust in competing‐risk survival analyses, and consistent patterns were observed across most subgroup analyses, with the exception of those stratified by race (Table 2 and Supporting Information S1: Figure S2). Among participants with morbidity, non‐White individuals had a substantially higher risk of HF compared with their White counterparts (Supporting Information S1: Figure S2).

**TABLE 2:** Association of the EASO classification and the extended EASO framework incorporating morbidity stratification with incident HF risk.

	Incident rate^a^	Model 1	Model 2	Model 3	Competing risk analysis
EASO					
Normal weight	1.31	Reference	Reference	Reference	Reference
EASO overweight	1.28	0.97 (0.92, 1.03)	0.82 (0.78, 0.87)	0.84 (0.80, 0.89)	0.81 (0.77, 0.86)
EASO new obesity	3.58	2.74 (2.61, 2.87)	1.41 (1.34, 1.48)	1.32 (1.25, 1.39)	1.43 (1.36, 1.51)
BMI obesity	4.04	3.09 (2.96, 3.23)	1.77 (1.69, 1.86)	1.62 (1.53, 1.71)	1.80 (1.72, 1.89)
EASO + morbidity (±)					
Normal weight (−)	0.88	Reference	Reference	Reference	Reference
Normal weight (+)	2.19	2.49 (2.32, 2.68)	1.68 (1.56, 1.80)	1.60 (1.49, 1.72)	1.59 (1.48, 1.71)
EASO overweight (−)	1.26	1.43 (1.34, 1.53)	1.03 (0.97, 1.11)	1.03 (0.96, 1.11)	1.03 (0.96, 1.11)
EASO new obesity (+)	3.28	3.74 (3.52, 3.98)	1.81 (1.70, 1.92)	1.67 (1.56, 1.78)	1.66 (1.55, 1.77)
BMI obesity (−)	2.01	2.28 (2.12, 2.46)	1.51 (1.39, 1.63)	1.44 (1.33, 1.56)	1.45 (1.34, 1.57)
BMI obesity (+)	5.84	6.40 (6.03, 6.80)	2.73 (2.56, 2.91)	2.37 (2.21, 2.54)	2.37 (2.21, 2.55)

*Note:* Model 1 was unadjusted. Model 2 was adjusted for age, sex, race, TDI, MET, diet score, tobacco and alcohol consumption, educational attainment, employment status, grip strength, and atrial fibrillation history. Model 3 was further adjusted for HDL, LDL, FPG, SBP, DBP, and eGFR. HRs and 95% CIs were estimated using Cox proportional hazards models. Competing risk analyses were performed using Model 3.

Abbreviations: BMI, body mass index; CI, confidence interval; DBP, diastolic blood pressure; EASO, European Association for the Study of Obesity; eGFR, estimated glomerular filtration rate; FPG, fasting plasma glucose; HDL, high‐density lipoprotein; HF, heart failure; HR, hazard ratio; LDL, low‐density lipoprotein; MET, metabolic equivalent of task; SBP, systolic blood pressure; TDI, townsend deprivation index.

^a^ Incidence rate per 1000 person‐years.

### 3.3 Extended EASO Framework Stratified by Morbidity and HF Risk

**FIGURE 3:**
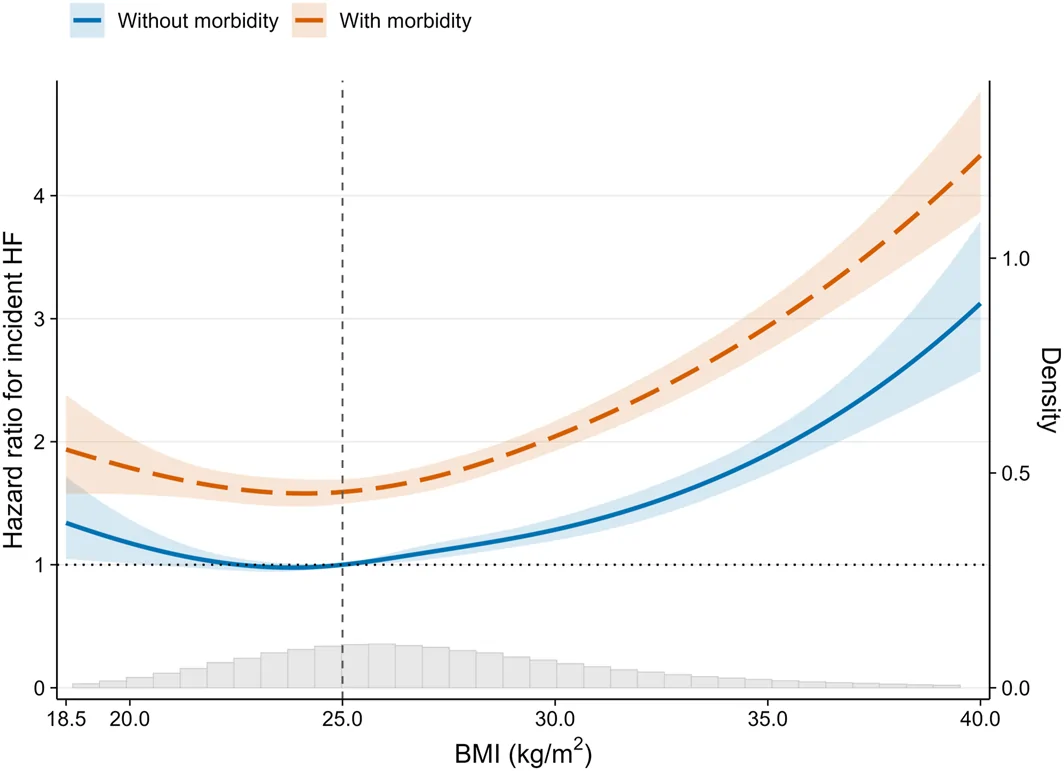
Association between continuous BMI and incident HF risk stratified by morbidity status. Hazard ratios were calculated relative to BMI = 25 kg/m^2^ among participants without morbidity, with covariates fixed at their mean or median values. BMI was modelled as a continuous variable using restricted cubic splines. HRs for incident HF across the BMI spectrum are shown for participants without morbidity (blue solid line) and with morbidity (orange dashed line). Shaded areas represent 95% confidence intervals. The grey histogram at the bottom displays the distribution of BMI in the study population. BMI, body mass index; HF, heart failure; HR, hazard ratio.

To address the heterogeneity of the conventional normal‐weight reference group and to extend morbidity stratification across all BMI categories, we applied the extended EASO framework using normal‐weight individuals without morbidity as the reference. This approach revealed that EASO overweight individuals without morbidity had HF risk statistically indistinguishable from the morbidity‐free normal‐weight reference (HR 1.03, 95% CI 0.96–1.11), indicating that overweight per se confers no excess HF risk in the absence of morbidity. By contrast, normal‐weight individuals with morbidity already exhibited markedly elevated risk (HR 1.60, 95% CI 1.49–1.72), comparable to that of EASO new obesity with morbidity (HR 1.67, 95% CI 1.56–1.78), underscoring that morbidity—rather than adiposity category—is the primary determinant of HF vulnerability in individuals with normal weight or overweight. Across all BMI categories, the presence of morbidity produced a consistent vertical upward shift in HF risk: BMI obesity without morbidity (HR 1.44, 95% CI 1.33–1.56) carried substantially lower risk than BMI obesity with morbidity (HR 2.37, 95% CI 2.21–2.54), and this morbidity‐driven risk gradient was observed uniformly regardless of adiposity level (Figure 2). Notably, normal‐weight individuals with morbidity (HR 1.60) exhibited higher HF risk than individuals with BMI obesity but without morbidity (HR 1.44), further demonstrating that morbidity burden supersedes anthropometric classification as a risk determinant. Incidence rates followed the same pattern, ranging from 0.88 per 1000 person‐years in normal‐weight individuals without morbidity to 5.84 per 1000 person‐years in BMI obesity with morbidity (Table 2). A similar dose–response relationship between BMI as a continuous variable and HF risk, stratified by morbidity status, was observed in restricted cubic spline analyses (Figure 3). These findings remained consistent across competing‐risk and subgroup analyses (Supporting Information S1: Figure S3).

### 3.4 Sensitivity Analysis Excluding Hypertension and CVD From Morbidity Definition

To address the potential circularity arising from the inclusion of hypertension and CVD—both strong independent HF risk factors—in the EASO morbidity definition, we repeated all analyses after excluding these two conditions. Results were materially unchanged across both frameworks (Supporting Information S1: Figure S4). In the EASO framework, EASO overweight showed no excess HF risk relative to normal weight (HR 1.01, 95% CI 0.97–1.06), whereas EASO new obesity retained a significantly elevated risk (HR 1.27, 95% CI 1.19–1.35) and BMI obesity remained the highest‐risk group (HR 1.59, 95% CI 1.50–1.67). In the extended framework anchored to normal‐weight individuals without morbidity, the vertical upward shift in HF risk conferred by morbidity was similarly preserved: normal weight with morbidity (HR 1.27, 95% CI 1.17–1.37), overweight without morbidity (HR 1.09, 95% CI 1.03–1.15), overweight with morbidity (HR 1.32, 95% CI 1.24–1.41), BMI obesity without morbidity (HR 1.56, 95% CI 1.46–1.65), and BMI obesity with morbidity (HR 2.03, 95% CI 1.90–2.18). These findings indicate that the morbidity‐driven risk stratification identified by the EASO and extended frameworks is not attributable to the inclusion of hypertension and CVD alone, and that the reclassification captures genuine HF vulnerability beyond that explained by these two conditions.

## 4 Discussion

In this large prospective population‐based cohort, we comprehensively evaluated the EASO obesity framework and its extended morbidity‐stratified version in relation to incident HF across the full BMI spectrum. Several key findings emerged. First, application of the EASO criteria substantially increased the estimated obesity prevalence compared with the BMI‐based classification. Although EASO overweight appeared low‐risk relative to the conventional normal‐weight reference, this comparison is confounded by the heterogeneity of the reference group, which includes individuals both with and without morbidity. When restricted to normal‐weight individuals without morbidity as the reference, EASO overweight showed no excess HF risk (HR 1.03, 95% CI 0.96–1.11), indicating that overweight per se confers no independent HF vulnerability in the absence of morbidity. Second, and most critically, morbidity status produced a consistent vertical upward shift in HF risk across all BMI categories in the extended framework: individuals with morbidity exhibited substantially higher HF risk than their morbidity‐free counterparts at the same adiposity level, and normal‐weight individuals with morbidity (HR 1.60, 95% CI 1.49–1.72) carried greater HF risk than individuals with BMI obesity but without morbidity (HR 1.44, 95% CI 1.33–1.56). These findings establish morbidity—rather than anthropometric classification alone—as an important determinant of HF risk across the full BMI spectrum. Third, these findings were robust in sensitivity analyses excluding hypertension and CVD from the morbidity definition, confirming that the morbidity‐driven risk stratification is not attributable to the inclusion of the two strongest independent HF risk factors alone.

Previous epidemiologic evidence has consistently demonstrated a nonlinear, frequently J‐shaped association between BMI and incident HF, with the lowest risk observed in the normal‐weight range [11, 12]. However, as a crude proxy of total adiposity, BMI does not adequately reflect variation in fat distribution, cardiometabolic burden, or underlying metabolic health [4, 13, 14, 15]. The EASO framework represents an important advance by refining risk stratification among individuals classified as overweight [7, 8]. In the primary EASO analysis, EASO overweight appeared to carry lower HF risk than the normal‐weight reference group (HR 0.84, 95% CI 0.80–0.89); however, this apparent protective association reflects the heterogeneity of the conventional normal‐weight reference, which aggregates individuals with and without morbidity. When the comparison is appropriately anchored to normal‐weight individuals without morbidity, EASO overweight shows no statistically significant excess risk (HR 1.03, 95% CI 0.96–1.11), indicating that the lower summary HR is a methodological artefact rather than evidence of a truly protective effect of overweight. Critically, this artefact arises precisely because the original EASO framework restricts morbidity stratification to the BMI 25–< 30 kg/m^2^ range, leaving the heterogeneity of the normal‐weight and obese groups unaddressed [7]. Our extended framework resolves this limitation by applying morbidity stratification across all BMI categories, revealing that the vertical upward shift in HF risk conferred by morbidity is a universal phenomenon independent of adiposity level. Metabolically adverse phenotypes are not confined to those with elevated BMI; normal‐weight individuals with morbidity carried markedly higher HF risk than obese individuals without morbidity, challenging the premise that anthropometric thresholds alone are sufficient for cardiovascular risk stratification.

The 2025 Lancet Diabetes and Endocrinology Commission proposed a complementary framework for obesity diagnosis that has received endorsement from more than 76 organisations [4]. Both the EASO and Lancet Commission frameworks share the fundamental principle that BMI alone is insufficient for clinical risk stratification and that phenotypic characterisation beyond anthropometric thresholds is required. The Lancet framework emphasises the distinction between clinical obesity—defined by objective signs of end‐organ dysfunction or impaired daily functioning attributable to excess adiposity—and pre‐clinical obesity, in which excess adiposity is present without current functional impairment. Our morbidity‐stratified findings are broadly concordant with this conceptualisation: the consistent vertical risk shift by morbidity across all BMI categories supports the Lancet Commission's emphasis on functional and clinical burden as the primary determinant of obesity‐related risk. However, both frameworks share a critical methodological consideration: the morbidities and functional impairments used to define clinical obesity severity are themselves established HF risk factors, introducing an inherent circularity in which the classification criteria and the outcome of interest are not fully independent. In the EASO framework specifically, conditions including hypertension, CVD, diabetes, CKD, COPD, depression, and arthritis define EASO new obesity, yet each independently predicts HF. This circularity is not unique to our study but is a structural feature of any morbidity‐integrated adiposity classification. To address this, we conducted sensitivity analyses excluding hypertension and CVD—the two strongest constituent HF risk factors—and found that the morbidity‐driven vertical risk shift was materially unchanged, suggesting that the stratification reflects a broader comorbidity burden rather than any single dominant condition. Whether the EASO or Lancet reclassification criteria add prognostic value for HF beyond the independent effects of their constituent morbidities remains an important question for future studies using Mendelian randomisation or interventional designs, and we acknowledge this as a limitation shared by both frameworks.

Our results have several important implications. First, our results support the adoption of the EASO framework as a clinically meaningful refinement of BMI‐based obesity classification for HF risk stratification, particularly by identifying a high‐risk phenotype within the conventionally defined overweight range that would otherwise be missed [16]. Second, our extended framework demonstrates that morbidity stratification should not be restricted to the overweight range but applied across the full BMI spectrum. The finding that normal‐weight individuals with morbidity carry greater HF risk than obese individuals without morbidity has direct clinical relevance: it suggests that HF prevention efforts and targeted surveillance should be guided by morbidity burden rather than BMI thresholds alone, potentially redirecting clinical attention towards high‐risk normal‐weight individuals who are currently overlooked by conventional screening approaches. Third, these findings support a broader paradigm shift from purely anthropometric toward phenotype‐based obesity classification in cardiovascular risk assessment. Routine clinical assessment of morbidity status across all BMI categories—rather than reserving intensive risk stratification for those with BMI ≥ 30 kg/m^2^—may improve the identification of individuals at elevated HF risk who could benefit from earlier lifestyle, pharmacologic, or monitoring interventions.

Several limitations should be acknowledged. The UK Biobank is predominantly composed of White participants, which may limit generalisability, although subgroup analyses suggested consistent patterns across most demographic strata [17]. Second, our operationalisation of EASO morbidity represents a pragmatic approximation of the full framework: conditions such as obstructive sleep apnoea, gastroesophageal reflux disease, urinary incontinence, and functional limitations are specified within the EASO criteria but were not fully capturable in UK Biobank, meaning that true EASO‐defined obesity prevalence is likely underestimated. Third, HF subtypes could not be formally examined in this cohort. Inpatient ICD‐10 subcodes such as I50.1, I50.0, and I50.9 do not directly encode the left ventricular ejection fraction and cannot establish definitive HFrEF/HFpEF classification, and a validated ICD‐based HF subtype algorithm was not available for this analysis. Cardiac MRI data were available in approximately 36,800 participants, but fewer than 80 incident HF cases had LVEF measured within one year of the HF event, including only four within 30 days. The limited event‐proximal imaging sample and substantial temporal mismatch between imaging and HF diagnosis precluded a meaningful MRI‐based subtype analysis. Whether the morbidity‐driven risk stratification differs between HF phenotypes warrants investigation in cohorts with echocardiographic or cardiac MRI phenotyping performed at or near HF diagnosis [18]. Fourth, residual confounding cannot be fully excluded despite extensive covariate adjustment, and the observational design precludes causal inference regarding the independent contribution of obesity reclassification versus comorbidity burden to HF risk. Finally, external validation of the extended EASO framework in independent cohorts with greater ethnic diversity and more comprehensive morbidity ascertainment is needed before these findings can inform clinical guidelines.

## 5 Conclusions

In this large prospective cohort, morbidity status emerged as a key determinant of HF risk across the full BMI spectrum, modifying the associations between adiposity classifications and HF outcomes. The extended EASO framework, by applying morbidity stratification across all BMI categories, revealed a consistent vertical upward shift in HF risk conferred by morbidity independent of adiposity level—a finding that challenges the primacy of anthropometric thresholds in cardiovascular risk stratification. These findings support a paradigm shift from BMI‐based toward phenotype‐based obesity classification, and highlight the importance of systematic morbidity assessment across the full BMI spectrum for early identification of individuals at elevated HF risk who may benefit from targeted prevention strategies.

## Author Contributions

Chenkai Wu and Zhenhua Xing conceived and supervised the study and conducted the data analysis. Liang Tang and Xianming Tang drafted the manuscript. All authors critically revised the manuscript and approved the final version.

## Funding

This work was supported by the Science and Technology Innovation Programme of Hunan Province (2024RC3062 to Zhenhua Xing), the Natural Science Foundation of Hunan Province (2023JJ30783 to Xianming Tang), and the Changsha Science and Technology Planning Project (kq2208313 to Xianming Tang).

## Ethics Statement

The UK Biobank has ethical approval from the North West Multi‐centre Research Ethics Committee. This study was conducted under an approved application using de‐identified data and was exempt from additional institutional review and informed consent.

## Consent

All authors approved the manuscript for publication.

## Conflicts of Interest

The authors declare no conflicts of interest.

## Supporting information


Supporting Information S1

## Data Availability

The data supporting the findings of this study are available from the UK Biobank (www.ukbiobank.ac.uk) under approved application procedures (Application No. 617672).
