# Supplementary material for: Redefining Obesity Risk for Heart Failure: Incorporating Morbidity Into the 2024 EASO Framework Definition of Obesity
Source: Diabetes Metab Res Rev. 2026 Sep 7;42(6):e70227. doi: 10.1002/dmrr.70227 (PMC13549250; doi:10.1002/dmrr.70227)
Supplement: Supplementary file 1 [file DMRR-42-e70227-s001.docx]

**Supplementary Table 1. Field IDs used to identify study outcomes and comorbities.**

|  | **Field IDs** |
| --- | --- |
| **HF** | 131355, 131354 |
| **All cause mortality** | 40020, 4000 |
| **HP** | 131287,131286 |
| **CVD** |  |
| Angina pectoris | 131297,131296 |
| AMI | 131299, [131298](https://biobank.ndph.ox.ac.uk/showcase/field.cgi?id=131298) |
| Stroke | 131369,131368 |
| **Diabetes** | 130709, 30708 |
| **COPD** | 42017, 42016 |
| **Depression** | 130895,130894 |
| **Arthritis** | 120000 |

*For each outcome or comorbidity, the first Field ID indicates whether the event , and the second Field ID records the date of occurrence. Time-to-event calculation: Event time was calculated as the interval between the event date (second Field ID) and the enrollment date. Participants without an event were censored at the end of follow-up (September 30, 2024).*

*Definition of all-cause mortality:*

*– For all-cause mortality, the event time was defined by the recorded death date; participants without a death record were censored at September 30, 2024.*

*– Arthritis was identified using UK Biobank Field ID 120000.*

*Abbreviations: HP, hypertension; CVD, cardiovascular disease; AMI, acute myocardial infarction; COPD, chronic obstructive pulmonary disease.*

**Supplementary Figure 1. Flow chart of present study**

*Abbreviations: BMI, body mass index; HF, heart failure; WtHR, waist-to-height ratio.*

**Supplementary Figure 2. Associations between EASO-defined obesity levels and HF, stratified by age, sex, and race**

*Note: The base model was adjusted for Model 3*

*Abbreviations: HF, heart failure; BMI, body mass index; EASO, European Association for the Study of Obesity.*

**Supplementary Figure 3. Association of EASO-defined obesity levels with HF by morbidity status, stratified by age, sex, and race.**

*Note: The base model was adjusted for Model 3*

*Abbreviations: HF, heart failure; BMI, body mass index; EASO, European Association for the Study of Obesity.*

**Supplementary Figure 4. Sensitivity analysis of associations between obesity categories and incident HF after excluding hypertension and CVD from the morbidity definition.**

Circles indicate groups without morbidity, and diamonds indicate groups with morbidity. In this sensitivity analysis, morbidity was redefined after excluding hypertension and CVD, and was based on arthritis, COPD, diabetes, CKD, or depression. The EASO classification used normal weight as the reference group, whereas the extended EASO framework used normal weight without morbidity as the reference group. Models were adjusted according to Model 3.

Abbreviations: HF, heart failure; EASO, European Association for the Study of Obesity; BMI, body mass index; CVD, cardiovascular disease; COPD, chronic obstructive pulmonary disease; CKD, chronic kidney disease; HR, hazard ratio; CI, confidence interval.
